# Metabolomic Diversity and Identification of Antibacterial Activities of Bacteria Isolated From Marine Sediments in Hawai’i and Puerto Rico

**DOI:** 10.3389/fmolb.2020.00023

**Published:** 2020-02-25

**Authors:** Ruhnaz Kashfi, Charles Kelsey, David Jorgen Gang, Douglas R. Call, David R. Gang

**Affiliations:** ^1^Institute of Biological Chemistry, Washington State University, Pullman, WA, United States; ^2^Department of Chemistry, Washington State University, Pullman, WA, United States; ^3^Paul G. Allen School for Global Animal Health, Washington State University, Pullman, WA, United States

**Keywords:** marine sediments, secondary metabolites, Actinobacteria, *Streptomyces*, *Bacillus*

## Abstract

Antibiotic resistance is a growing concern worldwide and consequently metabolomic tools are being applied increasingly in efforts aimed at identifying new antimicrobial compounds. Marine bacteria-derived compounds have shown great promise in this area. A metabolomics-based study was undertaken to study the diversity of secondary metabolites from marine sediment bacteria isolated from different locations of Hawai’i and Puerto Rico. This effort included characterizing the biodiversity in the sediment samples and searching for antibacterial activity and associated compounds. Bacterial strains were isolated using several different nutrient agars and culture conditions. DNA sequencing (16s rDNA) was used for phylogenetic characterization. Antibacterial activity was assessed against antibiotic-resistant strains of *Escherichia coli*, *Salmonella enterica*, *Acinetobacter baumannii*, *Staphylococcus aureus*, and *Enterococcus faecalis*. Ethyl acetate extracted bacterial secondary metabolites were measured by ultra-performance liquid chromatography-mass spectrometry, processed in Progenesis QI and further analyzed by partial least squares-discriminant analysis using MetaboAnalyst 3. Among the strains (*n* = 143) that were isolated from these two geographical areas and tested for antibiotic activity, 19 exhibited antibacterial activity against at least one antibiotic-resistant human pathogen. One strain from Hawai’i possessed broad-spectrum activity against all five pathogens. Metabolite profiles were diverse and separated the strains into two clusters in PCA analysis that mirrored geographical origin of the isolated strains. A diversity of bacteria and potential antibacterial compounds were observed in this study. Marine environments represent an opportunity to discover a rich diversity of antibacterial compounds for which resistance mechanisms may be uncommon in human pathogens.

## Introduction

Development of antibiotic resistance in pathogenic bacteria is a serious and growing health concern that necessitates development of new antibacterial drugs (Tambic Andrasevic, 2004; Bredholt et al., 2008). Application of metabolomics-based approaches to this problem may lead to more rapid advances in drug lead identification. One demonstrated source of antibiotics includes the gram-positive bacteria, such as Actinomycetes from the phylum Actinobacteria and *Bacillus* species from the Firmicutes, which are well known for producing important bioactive secondary metabolites including antibiotics (Stackebrandt et al., 1997; Berdy, 2005), antitumor agents (Feling et al., 2003; Cragg et al., 2011), and immunosuppressive agents (Mann, 2001), as well as useful enzymes (Ball and Mccarthy, 1988; Oldfield et al., 1998).

Most bacteria known to produce antimicrobial compounds have been isolated from terrestrial environments, but fewer novel compounds are being discovered in recent years (Shoichet, 2013; Wright, 2014). Actinomycetes and Bacillus species also inhabit a multitude of marine environments including mangrove forests (Hong et al., 2009; Lee et al., 2014), marine sponges (Kim et al., 2005; Kim et al., 2006), seaweed (Lee et al., 2008), and marine sediments (Mincer et al., 2002; Jensen et al., 2005; Bredholdt et al., 2007; Duncan et al., 2014). Among the Actinomycetes, *Streptomyces* species are the most well known for producing antibacterial compounds, but other antibiotic producing marine bacteria have been identified (Fiedler et al., 2005). Importantly, the metabolomic diversity among these organisms has not been investigated, and thus a large untapped resource remains to be examined using modern approaches.

The majority of marine bacteria investigations have focused on targeted compounds, but the extent of bacterial chemodiversity and presumably richness of antibacterial compounds is unclear. Depending on the scale of diversity across spatial gradients, multiple samples from relatively small geographic but physiographically diverse areas may yield a large number of candidate compounds for any particular drug lead question. The subtropical environments of Hawai’i and Puerto Rico share many similarities with regards to mean water temperature and latitude, but otherwise represent a large range of sediments, freshwater influence and human impact, and thus appear to contain a large range of diverse ecological niches that may support differential bioactive compound production among the marine microbes that inhabit these locales. Consequently, we investigated the metabolomic diversity and antibacterial activities of marine bacterial strains isolated from locations in Hawai’i (*n* = 7) and Puerto Rico (*n* = 19). Diverse strains, many new and unique, were identified that also possessed novel metabolomic profiles and diverse bioactivity profiles. One strain was particularly interesting owing to both its novel metabolomic profile compared to the other strains and to its potent antibacterial properties against major human pathogens.

## Materials and Methods

### Sampling of the Marine Sediments

Natural marine sediments (typically sand or finely crushed shell material present in environment in their native state) were collected around the Big Island of Hawai’i (Supplementary Figure S1A) and Puerto Rico (Supplementary Figure S1B) either from the intertidal zone on ocean beaches or at depth during scuba dives in areas where sediments were present within near-shore reefs. Supplementary Tables S1, S2 list all collection locations in Hawai’i and Puerto Rico, respectively, and indicate what type of sediment was collected (intertidal beach sand, underwater reef sediment, etc.) and its properties (color, grain size). Sediments were collected into sterile 50 ml conical tubes (Falcon-type), retaining a small amount of the collection site’s sea water to keep the sediment submerged after collection. In the case of beach sand, sand was collected where the surf was actively wetting the sand, but where sand could be collected between waves. Collection from start of the process to the finish thus typically took less than 10 s. Surface sand (the top 5 – 15 cm) was quickly scraped away using the pointed (bottom) end of the conical tube to expose the underlying sand, which remained wet from the sea water. The underlying sand was collected directly into the sterile conical tube, using the tube as a scoop. The cap of the tube was initially removed only immediately prior to using the tube to scoop up the underlying sand and then replaced immediately thereafter, to prevent contamination. No other tool besides the tube itself was used for each sand collection, preventing the possibility of cross-contamination from site to site. For the reef sediments, scuba-supported dives were performed by the corresponding author to enable collection of sediments from undersea locations, as listed in Supplementary Tables S1, S2. A similar collection process was followed for the underwater collections as for the intertidal zone collections: sterile tubes were used to scrape away the surface sediment and then the tube was opened immediately prior to collection of the sand using the tube again as a scoop. All samples were kept cool until they could be sent or taken to the laboratory: in a cooler until the end of the day, then in a refrigerator (∼4°C) until shipment or transport to the laboratory at the end of the collection trip. Sediment samples were stored at 4°C in their original collection tubes until used for strain isolation. We found that such samples can be stored for months or longer and still generate viable marine bacteria strains.

### Isolation of Bacterial Strains

Bacteria from marine sediments were dislodged by sonication using a Virsonic 475 ultrasonic cell disrupter system with a 20-kHz sonic probe at 109 μm amplitude. Sonication was chosen as a method as it dislodges more bacterial strains compared to a vortexing-based method (see below). On average (from 11 samples tested in an initial method development screen) 3.5 ± 1.0 and 13.1 ± 1.7 (mean ± SEM) unique strains were isolated, respectively, using the vortexing alone and vortexing plus sonication methods, with the total number of unique isolates per sample ranging from 0 to 24, depending on the sample. Therefore, the latter method (utilizing sonication) was selected as the method of choice for all additional effort. The mass of 1 cm^3^ wet sand/sediment was about 1.6 g. A number of parameters were evaluated to determine optimal conditions for bacterial dislodgement from the sediments. Triplicate sediment samples (sample size varied from 0.25, 0.5, 1.0, 1.5, or 2 cm^3^) were placed into 50 ml sterile plastic vials and mixed with 5 ml particle-free artificial sea water. The sonic probe was inserted into the sediment, with sonication proceeding for variable amounts of time (3, 30, 45, 60, 90, 180, and 360 s). Each sonication period was divided into 3 equal periods of sonication with at least 30 s intervals between them (3 × 1, 3 × 10, 3 × 15, 3 × 20, 3 × 30, 3 × 60, and 3 × 120 s). For prolonged sonication periods (>30 s) vials were placed in an ice bath during sonication to prevent overheating, which maintained the sample temperature under 20°C. The sediments were vigorously shaken by hand after sonication and allowed to settle for 5 s before the supernatant was collected. Sediments were extracted 2–4 more times as above. All 3 + supernatant fractions were combined and lightly centrifuged (500 × g, 5 min) and the clarified supernatant contained target bacteria (Epstein and Rossel, 1995). Optimum bacterial dislodgment was observed at 180 s and with smaller sample sizes (0.25 and 0.5 cm^3^), so we selected 180 s and 0.25 cm^3^ sample size for all further sample bacterial dislodgments.

Bacterial solution samples (marine sediment extracts) were plated on different nutrient agar plates using a sterile loop. Media used were M1, ISP2, AIA (all from Difco) and prepared following the exact protocol, including pH adjustment, indicated on the label. When rifampicin was added to the medium it was indicated by “R” in the sample label and supplemented at 100 μl⋅L^–1^ after the media had been autoclaved and was cool enough to touch. Inoculated agar plates were incubated at 27 and 37°C and bacterial growth was followed for 6–8 weeks. Many of the bacterial strains were very slow growing. Bacterial colonies were selected based on morphology and pigmentation produced. Single colonies were transferred to fresh agar plates and re-incubated to ensure growth of the pure strain. The plates were stored for several months at 4°C for medium term storage. Once the growth conditions were finalized for each strain, liquid cultures of each strain were grown in an orbital incubator shaker at 220 rpm, either at 27 or 37°C, depending on the growth temperature requirement of the particular strain, until the OD_600_ reached 0.7. Glycerol stocks were then generated (25% glycerol in solution) for long term storage at –80°C.

### Identification of Bacterial Strains

Genomic DNA was extracted from bacterial colonies using the CTAB method described by the Joint Genome Institute (Swarts et al., 2015). The protocol was followed as described except chloroform:isoamyl alcohol (24:1) was used at step 19 instead of phenol. Extracted DNA was used in a PCR reaction with 16S rDNA primers S-C-Act-235-a-S-20 (5′-CGCGGCCTATCAGCTTGTTG-3′) and S-C-Act-878-a-A-19 (5′-CCGTACTCCCCAGGCGGGG-3′) (Stach et al., 2003). For each reaction, a total volume of 10 μL was prepared using 1 μL of extracted DNA, 1 × Herculase II reaction buffer, 25 mM dNTPs, 0.5 μL Herculase II fusion DNA polymerase, 2% DMSO, and 0.25 μL of each primer (200 nmol ea.). PCR reactions were started with an initial denaturation at 95°C for 4 min followed by 38 cycles of 95°C for 20 s, 55°C for 20 s and annealing at 72°C for 30 s, with a final extension at 72°C for 3 min. PCR products were examined by 1% gel electrophoresis to determine the size of the products. PCR products were sequenced by a commercial vendor (Eurofins Genomics). After manual curation of sequence trace files, 380-base long fragments of 16S rRNA sequence were aligned and analyzed by BLASTn (Johnson et al., 2008) to identify Genbank sequences with the closest percent base identity. Sequences from this study of sufficient length and passing QC checks (including chimera checking) were submitted to GenBank under accessions MH393412 – MH393436.

### Antimicrobial Activity Testing

Bacteria used for testing were recovered from collections at Washington State University and included *Escherichia coli* (multi-locus sequence type 131),*Salmonella enterica* serovar Typhimurium Phage Type DT104,*Acinetobacter baumannii*, methicillin resistant *Staphylococcus aureus* and *Enterococcus faecalis.* These bacterial species were selected based on the Center for Disease Control’s list of bacterial strains that present imminent and potential threats of developing antibiotic resistance and included both Gram-negative and Gram-positive pathogens that are important nosocomial (*E. coli*, *A. baumannii*, *S. aureus*, and *E. faecalis*) and foodborne pathogens (*E. coli* and *S. enterica*) that can cause both gastroenteritis and systemic infection. Each pathogenic strain was grown overnight in Luria-Bertani liquid medium (LB, Hardy Diagnostics, Santa Maria, CA, United States), the OD was adjusted to 0.7, and then each culture was spread onto a Mueller Hinton agar plate to grow a “lawn” of bacteria (“test plates”). Marine bacterial strains were grown in liquid cultures as described in the Online Methods (Online Resource EM-1) for an extended period of time to induce them to produce secondary metabolites. Each bacterial strain was grown (7–14 days, depending on growth rate) in a liquid culture medium and appropriate temperature (27 or 37°C) to allow formation of the secondary metabolites, which are typically formed after the normal growth period. Cell cultures were centrifuged (21,000 × *g* at 4°C for 10 min) to separate the cells and cell debris from the medium and to generate CFSs. In addition, ethyl acetate extracts from each strain were generated as described below. Both the CFS and ethyl acetate fractions for each marine bacterial sample were then used in assays to determine their ability to inhibit growth of pathogenic bacteria. Ethyl acetate was chosen as an extraction solvent due to its moderate hydrophobicity and capability of extracting a large number of metabolites, excluding extremely non-polar and polar compounds. The latter would be present in the CFS. The CFS and ethyl acetate samples (10 μl of each) were spotted onto the prepared test plates. Plates were then incubated overnight (37°C, stationary incubator, ambient air). After 24 h, plates were examined for zones of inhibition that are indicative of growth inhibition associated with active compounds. Liquid iodine was used as a positive control for growth inhibition. Ethyl acetate when applied alone in this manner as control had no inhibitory effect on the pathogenic bacteria.

### Extraction of Bacterial Secondary Metabolites and UPLC-MS/MS Analysis

All solvents used for extractions and subsequent analyses were of liquid chromatography-mass spectrometry grade. Five biological replicates for each strain were grown in liquid cultures as outlined above. Ethyl acetate extracts for each strain were generated as follows: 600 μl of liquid culture were extracted with 2 × volume of solvent in a 2 mL Eppendorf tube, vortexed for 30 min and followed by 5 min of sonication in an ice bath to prevent overheating. The samples were then centrifuged at 21,000 × *g* at 4°C for 10 min and the cleared supernatants containing the extracted metabolites were transferred to fresh microfuge tubes. A small amount of the ethyl acetate fraction (10 μL) was removed for the bioassays as described above, with the rest being lyophilized and resuspended in 1:1 ACN: dd H_2_O with 1% formic acid. These samples were centrifuged at 14,000 × *g* for 4 min and the supernatants were transferred to vials for injection into the UPLC (ultra-performance liquid chromatography) instrument (Waters ACQUITY UPLC system; Waters Corporation, Milford, MA, United States) with an inline photo diode array (PDA) detector (UV/Vis range of 210–400 nm collected) that was connected to a Waters Synapt G2-S HDMS quadrupole–ion mobility spectrometry-time of flight mass spectrometer. For each sample, 1 μL was injected through a 2 μL sample loop using partial loop injection mode onto a Waters ACQUITY UPLC column (BEH C18, 1.7 μm, 2.1 × 50 mm), with a flow rate of 0.35 ml⋅min^–1^ with 0.1% formic acid in water (A) and 0.1% formic acid in acetonitrile (B) as solvents. A gradient elution profile was applied as follows: 95% A:5% B for the initial 2.2 min, gradient to 75% A:25% B at 4.5 min, gradient to 70% A:30% B at 6 min, gradient to 60% A:40% B at 12 min, gradient to 1% A:99% B at 16.5 min, return to initial conditions of 95% A:5% B at 19 min. The total analysis time was 19 min. The autosampler and column temperatures were kept at 8°C and 30°C, respectively.

The Synapt G2-S HDMS Q-IMS-TOF was operated in both ESI positive and negative ionization modes and in the resolution mass mode [full width at half maximum (FWHM) of ∼40,000] with lockspray and without using the ion mobility features of the instrument. Due to far fewer compounds being detected in the negative ion mode in initial test runs, only the positive ion mode was used for data acquisition for the metabolomics-based screening procedure outlined in this manuscript. This in no way suggests that the few metabolites observed in negative mode are insignificant. Indeed, the actual active compounds present in the active strains may be better (perhaps only) detected using negative mode. However, purification and identification of the active principles is beyond the scope of this article and will be pursued in follow-up research that seeks to discover the actual identity of those active compounds. Mass spectral data were collected in both full MS and MS^e^ modes, with the latter using a medium collision energy ramp (20–30 V) for fragmentation, with all other parameters being identical between the two scan modes as follows. The scan range was from 100 to 2000 *m/z* with a scan time of 0.3 s. The capillary voltage, sampling cone voltage, and source offset voltage were 3.0 kV, 35 V, and 80 V, respectively. The source temperature was 100°C with a cone gas (nitrogen) flow rate of 20 L⋅h^–1^. The desolvation temperature was 350°C with a desolvation gas (nitrogen) flow rate of 600 L⋅h^–1^. The nebulizer gas (nitrogen) flow was 6.0 bar and leucine enkephalin used as the lock mass compound with a reference mass of *m/z* 556.2771. Data were analyzed using vendor software (MassLynx), Progenesis QI and MetaboAnalyst, using default parameters. The raw data supporting the conclusions of this article will be made available by the authors, without undue reservation, to any qualified researcher.

To reduce the number of metabolite features used in the different downstream analyses, to narrow the focus to metabolites potentially unique to specific strains (also to eliminate metabolites common to all strains), and to eliminate multiple adduct species for the same compound, acceptance criteria were applied. These included: (i) *m/z* error of <5 ppm compared to theoretical mass of potential formula, (ii) retention time error of <2% compared to the defined retention time for known or already detected compounds, (iii) low *p*-values (<0.05), and (iv) identification based on elemental composition and searches against the ChemSpider, PubChem and KEGG databases. For analyses performed using Metaboanalyst v. 3, the data were also normalized using IQR normalization, log transformation and Pareto scaling. Application of these acceptance criteria led to a reduction in metabolite features compared across samples from the initial ∼3800 detected to 678 putative compounds (likely to be unique, real compounds and not adducts or noise) that differed significantly in abundance between at least two of the strains (based on peak area, *p* < 0.05).

## Results

### Isolation and Identification of Bacterial Strains

**FIGURE 1:**
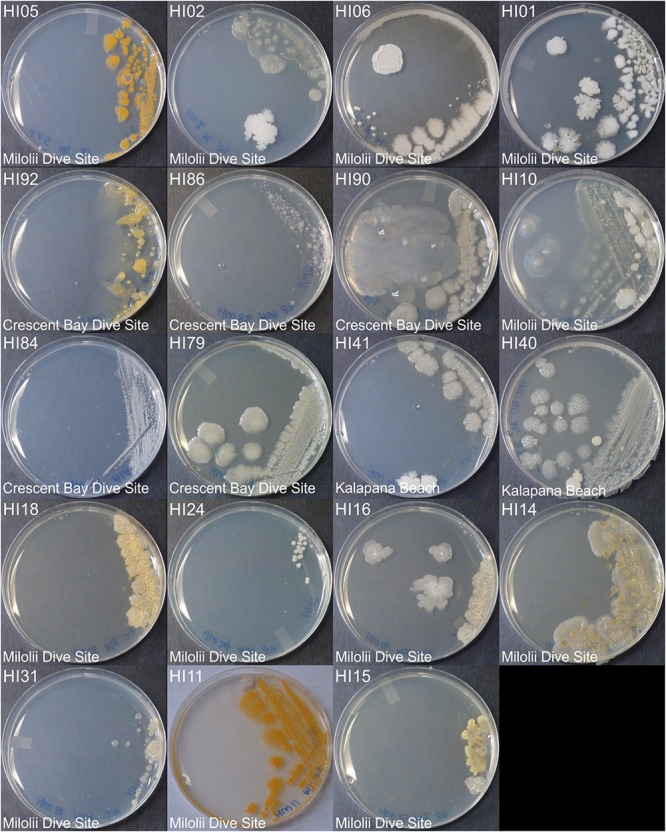
Representative bacterial colonies with different morphologies growing on agar plates. Samples are from the top cluster in Figure 3.

**FIGURE 2:**
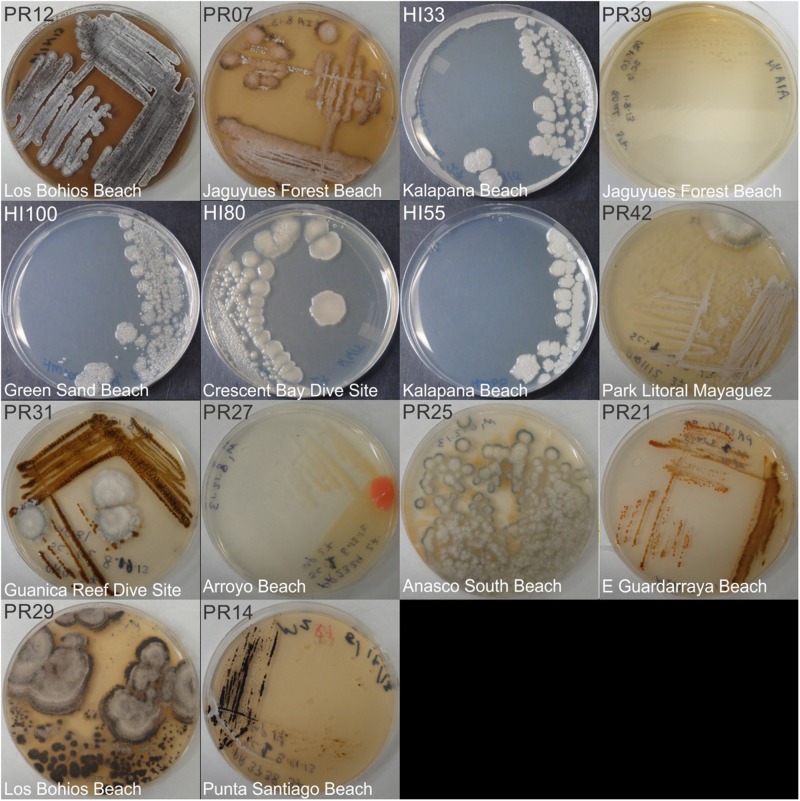
Representative bacterial colonies with different morphologies growing on agar plates. Samples are from the bottom cluster in Figure 3.

**FIGURE 3:**
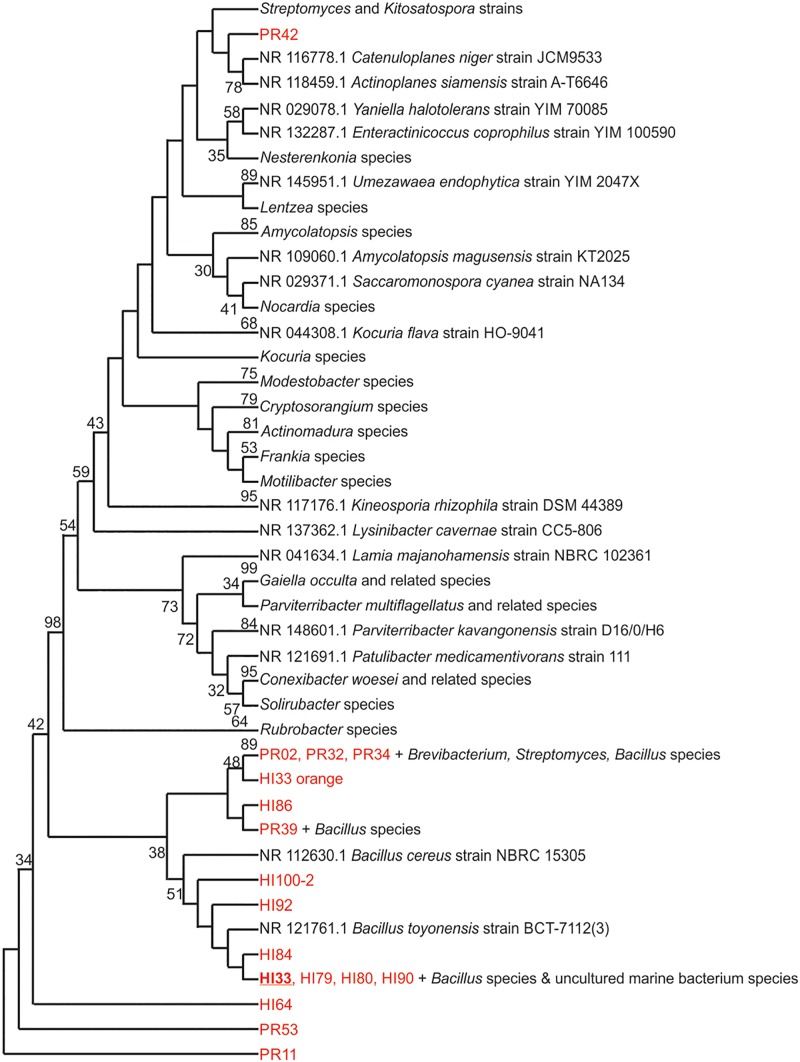
Evolutionary relationships of bacterial strains were analyzed. The evolutionary history was inferred using the Neighbor-Joining method (Saitou and Nei, 1987). The bootstrap consensus tree inferred from 500 replicates (Felsenstein, 1985) is taken to represent the evolutionary history of the taxa analyzed. Branches corresponding to partitions reproduced in less than 50% bootstrap replicates are collapsed. The percentage of replicate trees in which the associated taxa clustered together in the bootstrap test (500 replicates) are shown next to the branches (Felsenstein, 1985). The evolutionary distances were computed using the Maximum Composite Likelihood method (Tamura et al., 2004) and are in the units of the number of base substitutions per site. The analysis involved 125 nucleotide sequences. All positions containing gaps and missing data were eliminated. There were a total of 153 positions in the final dataset. Evolutionary analyses were conducted in MEGA7 (Kumar et al., 2016).

Marine bacteria were isolated from seven (*n* = 84 strains) and nineteen (*n* = 59 strains) different locations in Hawai’i (Big Island, Supplementary Figure S1A) and Puerto Rico (Supplementary Figure S1B), respectively. Examples of colony morphologies for strains with particular relevance (bioactivity, important positions in phylogenetic tree, see below) are show in Figures 1, 2. Eighteen strains were selected for sequence comparisons, including both those with significant antibacterial zones of clearance (see antibiotic activity section below) and others with very unique colony morphology. BLASTn searches indicated that the majority of the isolated strains belong to Actinobacteria and Firmicutes phyla with similarity to *Streptomyces, Motilibacter, Kitasatospora, Actinomadura, Cryptosporangium, Frankia, Kocuria, Micrococcaceae, Nocardia*, and *Bacillus*, such as *B. subtilis, B. amyloliquefaciens, B. nematocidal, B. anthracis, B. toyonensis*, and *B. cereus*. Phylogenetic analysis revealed a diverse collection of species (Figure 3), although most of the active strains were *Bacilli*, which was a bit surprising as the Actinobacteria are better known for diverse antibiotic activities. These species, especially HI33, will be the target of antibiotic development in future research (beyond the scope of this investigation).

### Bioactivity of Isolated Strains

**FIGURE 4:**
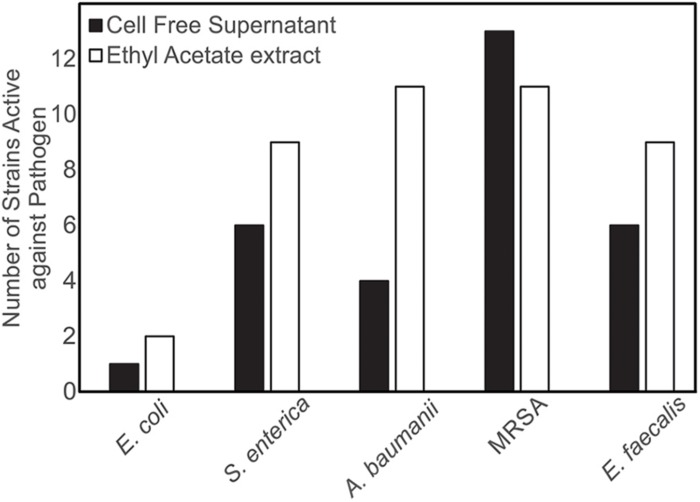
Isolates of marine bacteria (*n* = 143) were used to prepare cell-free supernatants (CFS) and ethyl acetate (EA) extracts. These materials were then assessed for antibacterial activity and the number of strains with visible zones of clearance associated with the CFS and/or EA were tallied.

Both CFSs and ethyl acetate extracts (EA) from each strain were tested against five pathogenic bacteria as outlined in section “Materials and Methods.” These fractions (CFS and EA) were chosen for analysis due to the broad polarity range (very polar for CFS and relatively non-polar for EA) of compounds that would be present in the two fractions. Because extracts, and not purified compounds, were used, we have not yet purified the active constituents, it was not possible to generate minimum inhibitory concentration (MIC) curves for the bioactivities observed in these strains. Nevertheless, EA samples were more likely to show activity against the test bacteria, based on the number of the different strains of the pathogenic bacteria that the EA vs. CFS fractions from a given isolated strain were active against (Figure 4). Several strains (19 out of 143) exhibited antibacterial activity, and the spectrum of activity was occasionally different for the CFS and EA preparations, as shown in Table 1 and Supplementary Tables S3–S7. For example, extractions from strain HI33 exhibited activity against all of the test bacteria. Other extractions exhibited activity against all species except for *E. coli* (e.g., HI02, HI05, HI10, etc.). Others were more specific to Gram-negative bacteria (HI06) or against individual species (PR07, PR09, HI84, HI86, HI90, etc.). For some strains, these activities were limited to either the CFS or the EA extract, whereas for others, both extracts exhibited antibacterial activity. Based on these results, we can predict that multiple classes of antibiotic compounds that target multiple mechanisms are present in this collection.

**TABLE 1:** Bacterial strains found with the strongest activity against at least one of the serious human pathogenic bacteria tested in the agar diffusion assays.

	***Escherichia coli***		***Salmonella enterica***		***Acinetobacter***		**MRSA**		***Enterococcus***	
	**ST-131**		**DT104**		***baumannii***				***faecalis* EPH 401**	
**Sample**	**CFS**	**EA**	**CFS**	**EA**	**CFS**	**EA**	**CFS**	**EA**	**CFS**	**EA**
HI05				X		X	X	X		X
HI02				X		X	X	X		X
HI06				X		X				
HI01		X	X	X	X			X	X	X
HI92							X	X		
HI86							X	X		
HI90							X			
HI10			X	X		X	X	X	X	
HI84							X			
HI79					X	X	X		X	X
HI16			X	X		X		X	X	X
HI14			X	X	X	X	X	X		X
HI11						X				X
HI15			X	X		X		X	X	X
PR07							X			
HI33	X	X	X	X	X	X	X	X	X	X
PR39							X			
HI100								X		
HI80						X	X			

*Samples are ordered according to relationships outlined in Figure 5. Cell free supernatant indicates results from assays where cell free supernatants (media free of cells) were tested. EA indicates ethyl acetate extracts were tested. X indicates activity in the form of a cleared zone on the agar plate around the region where the extract was spotted. Blank cells indicated no activity detected under assay conditions. HI, samples from Hawai’i; PR, samples from Puerto Rico.*

### Metabolomic Analysis

**FIGURE 6:**
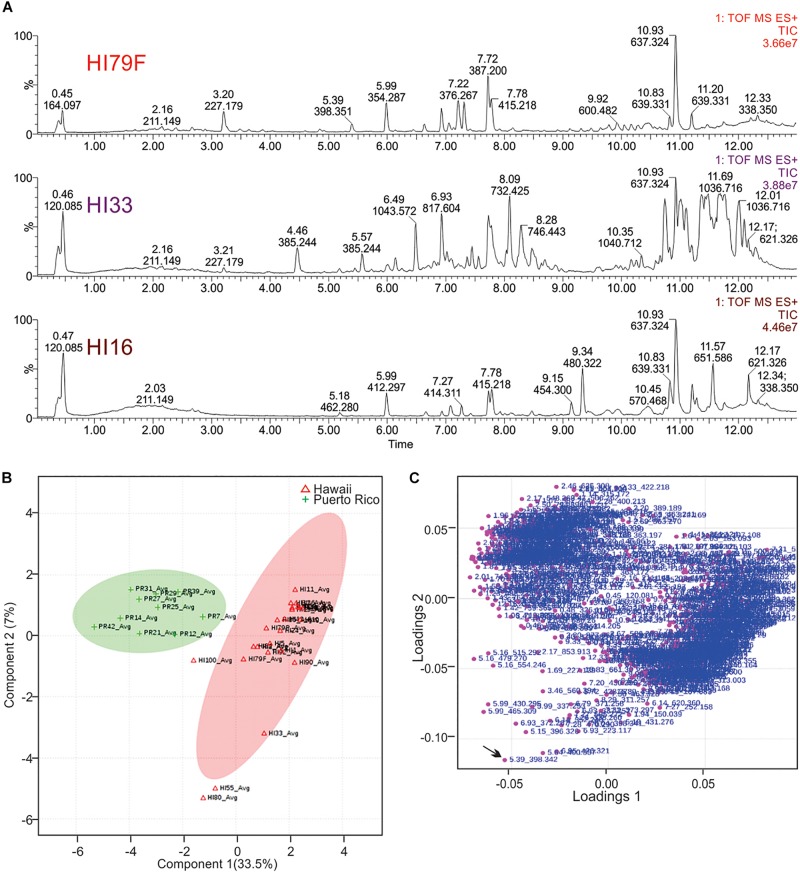
Principal Components Analysis of metabolites produced by strains from Hawai’i and Puerto Rico. **(A)** Total ion current (TIC) chromatograms from representative strains from Hawai’i. **(B)** PCA scores plot comparing strains from Puerto Rico and Hawai’i. The “Avg” suffix indicates that the “samples” displayed are the average values of all 5 replicates analyzed per strain. **(C)** Loadings plot from PCA analysis.

**FIGURE 5:**
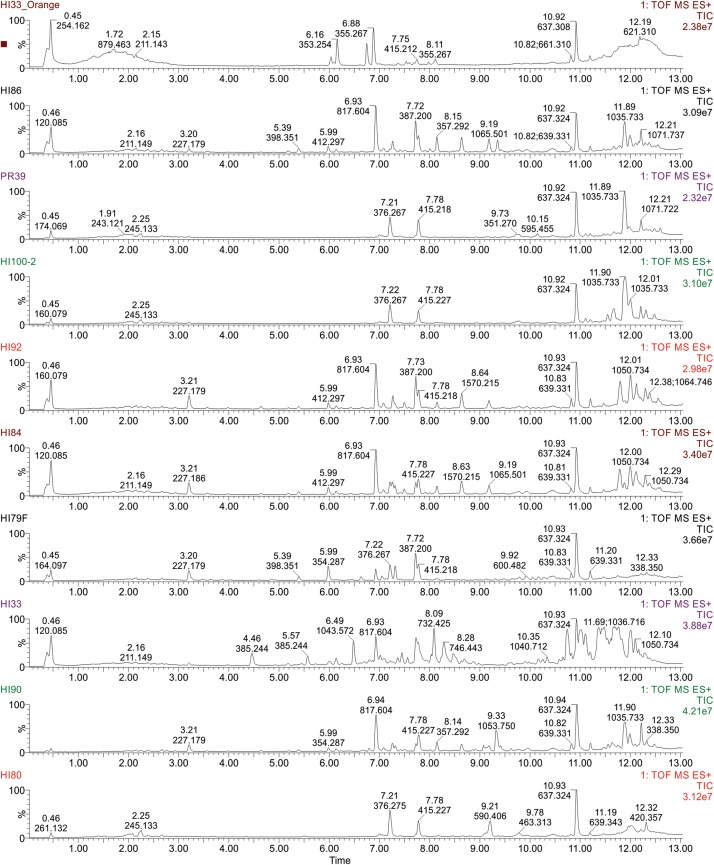
Ultra performance liquid chromatography-tandem mass spectrometry analysis of ethyl acetate extractable metabolites produced by strains from Hawai’i and Puerto Rico. Examples of TIC Chromatograms from Bacterial strains in the bottom cluster in the phylogenetic tree.

Ethyl acetate extracts from 33 strains were subjected to non-targeted UPLC-MS analysis on a Waters Synapt G2-S HDMS instrument, as outlined in section “Materials and Methods.” Strains were selected based on activity and unique colony morphology. Extracted metabolite profiles for each sample (mass range from 100 – 2000 *m/z* over the run window from 0.5 – 18 min) were generated (see Figure 5) and then analyzed using MassLynx, Progenesis QI and MetaboAnalyst software, which were used to detect over 3800 metabolite features in the combined dataset (see Figure 6). Ultimately, 678 putative compounds (condensed dataset) were determined to provide discriminative information and this condensed dataset was used for additional analyses. The other ∼3200 features did not differ significantly between strains, as determined by both the Progenesis QI and MetaboAnalyst software packages and appeared to represent common metabolites, such as membrane lipids, primary metabolites, some peptides, etc.

Unsupervised PCA analysis of the strain collection using this condensed dataset clearly separated strains from Hawai’i and Puerto Rico into two separate groupings (Figure 6B). Strain HI33, which had very evident and broad spectrum antibiotic activity, did not cluster with the other samples from Hawai’i or Puerto Rico. Strains HI55, HI80, and HI100 also appeared to be outliers. A relatively clear geographic separation in overall metabolome was evident despite many common genera between the two islands.

The loadings plot from PLS-DA analysis of the condensed dataset is shown in Figure 6C, where it is clear that most compounds have similar variances. A few, however, such as *m/z* 398.342 with retention time 5.39 min (in the lower left quadrant, indicated by an arrow), are more significant. The two large groupings of metabolites in the loadings plot (left and right sides of the graph) contain compounds present in extracts from Puerto Rico and Hawai’i, respectively.

**FIGURE 7:**
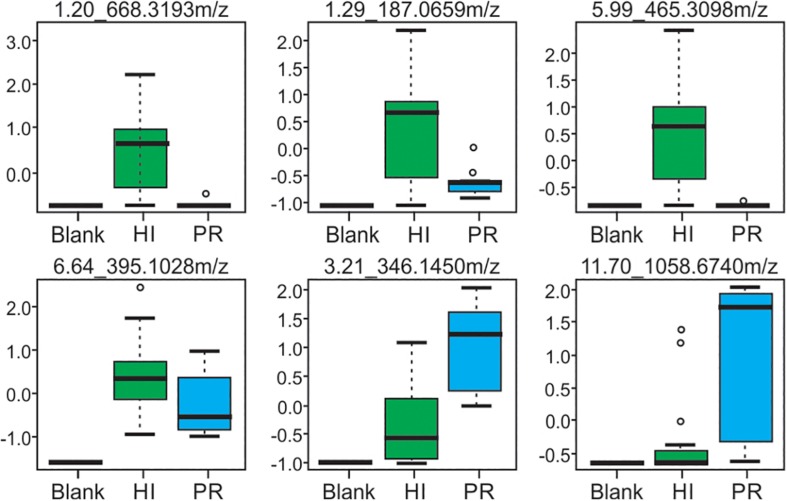
Box and whisker plots of selected metabolites produced by strains from both Hawai’i and Puerto Rico, demonstrating differences in abundance. Compounds are defined by their retention time and *m/z*-values as indicated at the top of each box.

**FIGURE 8:**
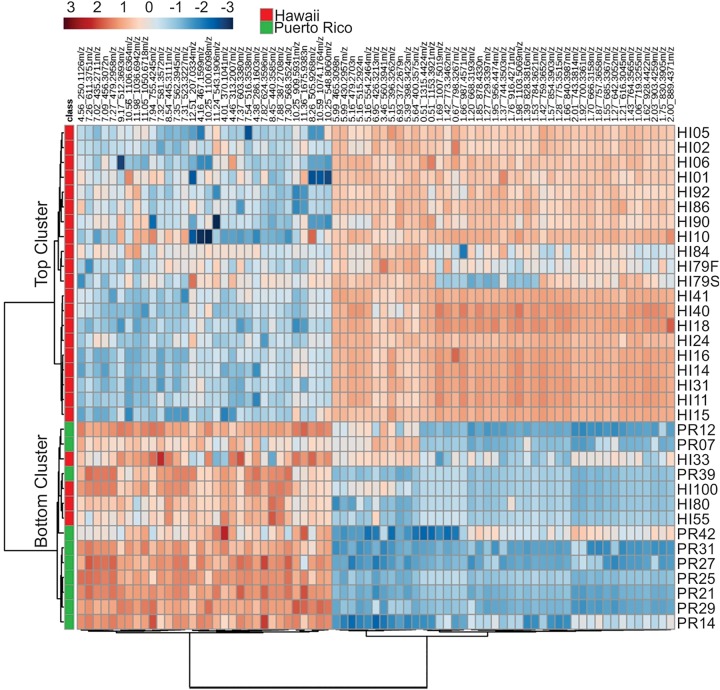
Cluster analysis of the 75 most influential metabolites (based on PLS-DA) and their relative abundances in the different strains analyzed.

**FIGURE 9:**
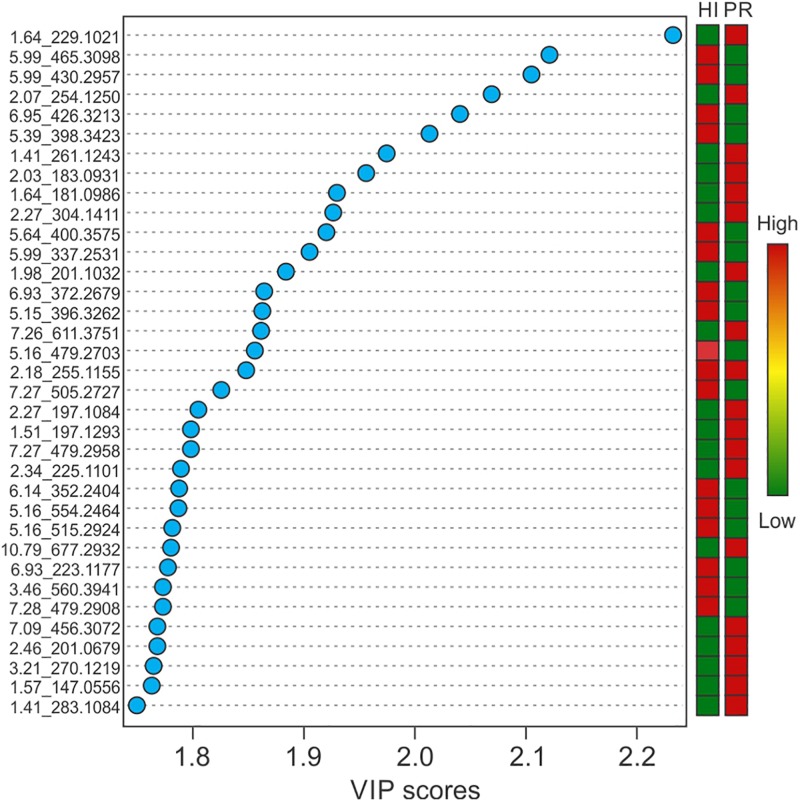
Variable importance in projection scores of the top 35 most impactful metabolites, based on PLS-DA, and their relative abundance by location.

Representative box and whisker plots (see Figure 7) illustrate some differences in the relative abundance of metabolites between geographic locations. A heat map was generated using hierarchical clustering analysis for the 75 metabolites that appeared to differ the most by geographic location (Figure 8). Difference in this case was based on the variable importance in projection (VIP) scores from a PLS-DA analysis (e.g., see Figure 9) among the 33 strains. Again, differences by geographic origin are clearly evident.

**FIGURE 10:**
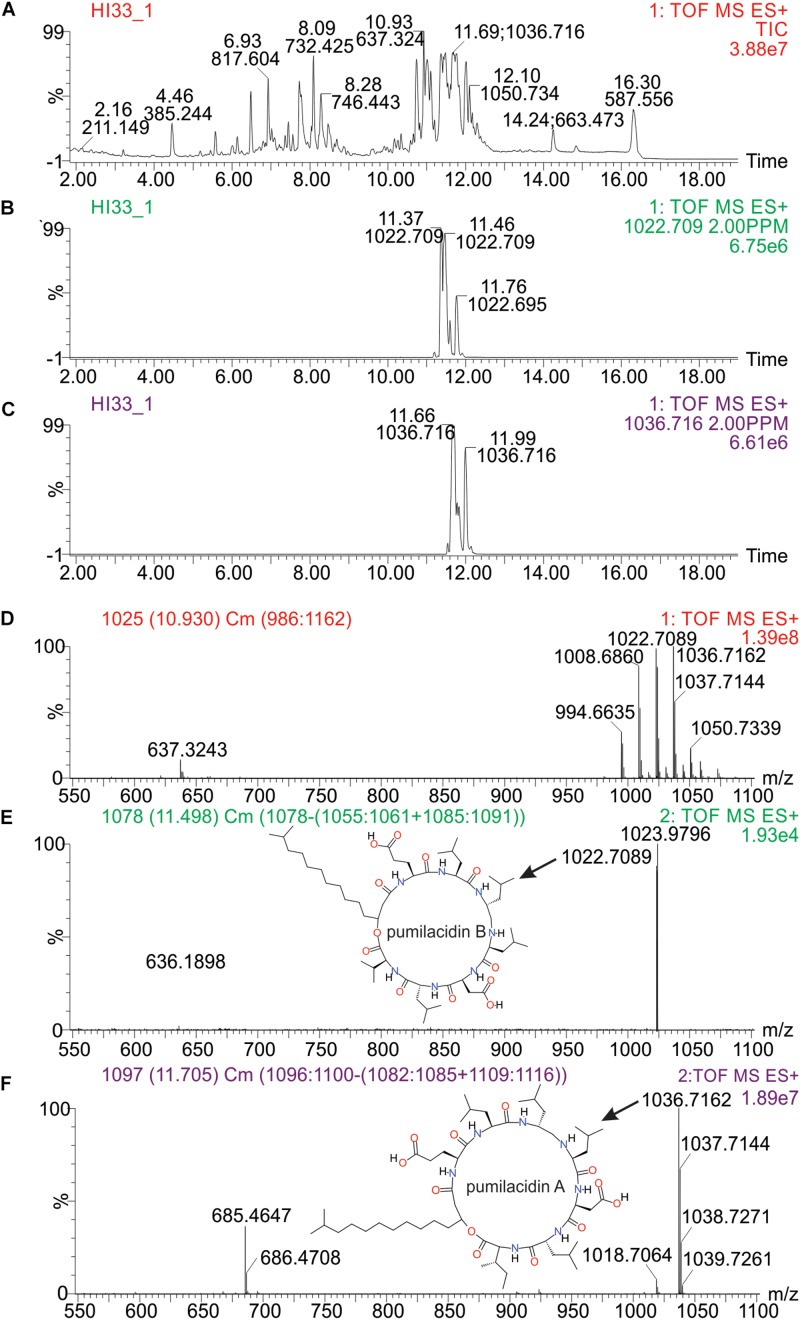
Analysis of specific metabolites from strain HI33, including: **(A)** total ion chromatogram from ESI + full scan MS analysis of the ethyl acetate extract from liquid cultures of the strain grown for an extended period as described in section “Materials and Methods,” **(B,C)** selected ion chromatograms for compounds with *m/z* 1022.709 and *m/z* 1036.716, respectively, **(D)** combined full mass spectrum for the compounds eluting between 10.5 and 12.5 min in the UPLC-MS analysis, and **(E,F)** the MS^E^ spectra for the peaks at 11.5 and 11.7 min in the UPLC-MS/MS analysis, respectively, including the structures of pumilacidin B and pumilacidin A (candidate compounds in this cluster, inset) collected from PubChem (Kim et al., 2016).

Molecular formulae were generated from accurate mass *m/z*-values for compounds that appeared particularly interesting in the PCA and PLS-DA analysis. Proposed compounds were selected based on statistical and manual curation and compared to the database in METLIN^1^ that matched the VIP scores with potential compounds (Supplementary Table S8). A few of the metabolites unique to HI33 were dereplicated using the AntiMarin 2013 database (Macintyre et al., 2014) and appeared to be related to pumilacidin peptides (similar to surfactins), which have been previously reported as metabolites from *Bacillus pumilus* (Naruse et al., 1990). Analysis of the chromatogram and metabolites produced by strain HI33 revealed a cluster of metabolites eluting between 11.5 and 12.3 min in the UPLC-MS/MS analysis (Figure 10) that were unique to this strain (see Figures 5, 6). The metabolites eluting in this range with *m/z* 1022.7089 and *m/z* 1036.7162, respectively, have the same mass as pumilacidin B or other cyclic peptide and pumilacidin A or other cyclic peptide, with predicted molecular formulae of C_53_H_95_N_7_O_12_ and C_54_H_97_N_7_O_12_. Pumilacidins exhibit antiviral and antibacterial properties (Naruse et al., 1990; Xiu et al., 2017), and thus this class of compounds is a reasonable first estimate as to the identity of some of the active compounds produced by the HI33 strain. Based on the broad spectrum of activity displayed (Table 1) against very different types of human pathogenic bacteria, however, it is clear that other compounds (as yet unidentified) produced by this strain have antibiotic activities as well.

## Discussion

Marine sediments from Puerto Rico and Hawai’i harbor a rich collection of bacteria with dramatic chemical (metabolomic) diversity and antibiotic activities. The Hawai’ian samples appeared to harbor greater bacterial diversity (at both the genetic and metabolomic levels) and more strains with antibacterial activity, based on the sampling performed in this investigation. Hawai’i and Puerto Rico are located at a similar latitude and have similar water temperatures year round: 25.0 – 27.8°C in Hawai’i and 25.0 – 30.6°C in Puerto Rico, although Hawai’i in general experiences less rainfall and wind flow compared to Puerto Rico, and of course one (Hawai’i) is in the middle of the Pacific Ocean and the other (Puerto Rico) sits at the boundary between the Atlantic Ocean and the Caribbean Sea. Differences in ocean water composition could contribute to differences in bacterial composition. A major difference between the islands is the volcanic activity present on Hawai’i, and the strain in our collection with the broadest spectrum activity was isolated from a relatively young black sand beach (Kalapana) that was formed after 1992 when Episode 48 (from Kupaianaha crater) of the Pu’u ‘O’o eruption of Kilauea ended^2^. Interestingly, this beach was selected as a potential “control” beach by us, as it was isolated, with no natural vegetation in the vicinity, and not being a popular location for sunbathing or swimming, and we had thought that it might therefore have a much more limited number of bacterial species associated with its sand. Obviously, this presumption was incorrect. Unfortunately, this beach has since disappeared under the latest lava flows from 2018. Thus, the discovery of this strain and its unique metabolomic and bioactive profiles within this short timeframe was fortuitous. This strain was not detected at any of the other sites sampled.

A large variation in secondary metabolite compounds was detected in the marine bacteria isolated from these two islands. Nevertheless, attempts to connect specific chemical profiles with antimicrobial activity did not detect any consistent patterns. Moreover, there was no consistent trend with regards to percentage of strains with antibiotic activity. For example, at the Milolii and Crescent Bay dive sites, 12 out of 20 and 6 out of 22 strains, respectively, possessed extractable compounds with antibiotic activity, whereas the Kalapana and Green Sand Beach sites had far lower percentages of active strains (1 out of 19 and 1 out of 12, respectively).

Also, contrary to our expectation of finding mostly actinobacterial species as active strains in these marine sediment samples, we observed many members of the Bacillales exhibiting potentially important antimicrobial activity. Species-level identification could not be confirmed at this time as most of these strains matched with less than 95% homology to any sequence in the NCBI database, indicating the potential that these are previously undescribed species. Thus, significant and novel chemodiversity was observed to be connected to new and novel marine bacterial strains/species. At the genus level, all of the genera detected in our analysis of samples from Hawai’i and Puerto Rico were previously isolated from marine environments in various regions of the world (Kämpfer, 2012; Brown-Elliott et al., 2015; Sharma et al., 2016), indicating that these genera are likely to be widely distributed in marine environments worldwide. Individual species, on the other hand, are likely to be limited to specific locations. Indeed, a large majority of the strains identified were found at only one of the collection sites, thus suggesting that a large, untapped resource is present in marine sediments around the globe.

## Conclusion

Marine sediments from two superficially similar environments produced a wide diversity of bacterial strains that were readily culturable in the laboratory and that possessed diverse metabolomes and antibiotic activities. A fraction of these strains (∼13%) exhibited antibacterial activity against antibiotic-resistant bacterial pathogens. Metabolomic analysis demonstrated that these strains were unique from other non-active strains and from each other, at the overall metabolome level. In addition, we detected unique and potentially important classes of secondary metabolites that are present in these strains and that may contribute to the observed antimicrobial activity. Further work will be needed to isolate, purify, fully characterize and synthesize the compounds with antibacterial activity.

## Data Availability Statement

The raw data supporting the conclusions of this article will be made available by the authors, without undue reservation, to any qualified researcher.

## Author Contributions

DRG conceived and designed the research. RK, CK, DJG, and DRG conducted the experiments. DC provided the cell lines, assisted with bioassay performance and editing the manuscript. RK and DRG analyzed the data and wrote the manuscript. All authors read and approved the manuscript.

## Conflict of Interest

The authors declare that the research was conducted in the absence of any commercial or financial relationships that could be construed as a potential conflict of interest.

## Footnotes

1 metlin.scripps.edu

2 https://www.soest.Hawaii.edu/GG/HCV/puuoo-episodes.html

## Abbreviations

CFS: cell free supernatant
CTAB: hexadecyltrimethylammonium bromide
EA: ethylacetate
HDMS Q-IMS -TOF: high definition mass spectrometry quadrupole-ion mobility spectrometry-time of flight
PCA: principal component analysis
PLS-DA: partial least squares-discriminant analysis
UPLC-MS/MS: ultra performance liquid chromatography-tandem mass spectrometry.
