# Supplementary material for: Metabolomic Diversity and Identification of Antibacterial Activities of Bacteria Isolated From Marine Sediments in Hawai’i and Puerto Rico
Source: Front Mol Biosci. 2020 Feb 25;7:23. doi: 10.3389/fmolb.2020.00023 (PMC7052060; doi:10.3389/fmolb.2020.00023)
Supplement: Supplementary file 2 [file Data_Sheet_1.pdf]

Table S8. Probable identification of the metabolites selected from the dataset based on VIP scores derived from the PCA and PLS-DA analysis and annotated after METLIN searches.

**Rt (min)**	**m/z**	**Molecular formula**	**Possible adducts**	**Most probable Metlin match**
1.41	261.1243	C14H18N2O4<br>C17H19NaO2<br>C14H16N2O3<br>C17H17NaO<br>C17H18O<br>C13H12N2O2<br>C16H13Na<br>C14H22O2<br>C12H13NO3	M+H-H2O<br>M+H-H2O<br>M+H<br>M+H<br>M+Na<br>M+CH3OH+H<br>M+CH3OH+H<br>M+K<br>M+ACN+H	C14H18N2O4<br>[M+H-H2O]
1.57	147.0556	C8H8N2O2<br>C8H6N2O<br>C7H2N2	M+H-H2O<br>M+H<br>M+CH3OH+H	C8H8N2O2<br>[M+H-H2O]
1.98	201.1032	C12H14N2O2<br>C15H15Na<br>C12H12N2O<br>C12H18	M+H-H2O<br>M+H-H2O<br>M+H<br>M+K	C12H12N2O<br>[M+H]
2.27	197.1084	C8H19N2NaOS<br>C8H17N2NaS<br>C8H18N2S	M+H-H2O<br>M+H<br>M+Na	C8H18N2S<br>[M+Na]<br>C10H13FN2O<br>[M+H]
2.46	201.0679	C14H11NaO<br>C14H9Na<br>C14H10	M+H-H2O<br>M+H<br>M+Na	C10H13ClO2<br>[M+H]
3.46	560.3941	C32H50N5NaO2<br>C32H49NO5<br>C33H45N5O<br>C30H50NNaO5<br>C31H46N5NaO<br>C31H50O6<br>C32H46N4O2<br>C30H47N4NaO2	M+H<br>M+CH3OH+H<br>M+CH3OH+H<br>M+CH3OH+H<br>M+CH3OH+H<br>M+ACN+H<br>M+ACN+H<br>M+ACN+H	C33H53NO6<br>*CAS:* 511-36-4<br>[M+H]
5.16	479.2703	C26H41NO5S<br>C24H42NNaO5S<br>C21H38N5NaO6<br>C25H38N5NaOS	M+H-H2O, M+H<br>M+H-H2O, M+H<br>M+H-H2O, M+H<br>M+H-H2O, M+H	C19H36N8O5<br>[M+Na]
5.16	515.2924	C22H45NO12	M+H-H2O<br>M+H<br>M+Na	C21H38N8O7<br>[M+H]
5.16	554.2464	C23H41NO15<br>C24H37N5O11<br>C21H41N5O11S	M+H-H2O<br>M+H-H2O<br>M+H-H2O	C24H35N5O10<br>[M+H]

		C19H42N5NaO11S<br>C23H39NO14<br>C20H43NO14S<br>C24H35N5O10<br>C21H39N5O10S<br>C19H40N5NaO10S<br>C19H41N5O10S<br>C22H42N3NaO8S<br>C22H35NO13<br>C19H39NO13S<br>C23H31N5O9<br>C20H35N5O9S<br>C18H36N5NaO9S<br>C24H41N3O9<br>C20H45N5O6S2<br>C22H42N3NaO9<br>C22H32N4O10<br>C25H33N2NaO8<br>C17H37N4NaO10S<br>C21H37N4NaO5S2<br>C22H43N3O8S<br>C20H44N3NaO8S	M+H-H2O<br>M+H<br>M+H<br>M+H<br>M+H<br>M+H<br>M+Na<br>M+Na<br>M+CH3OH+H<br>M+CH3OH+H<br>M+CH3OH+H<br>M+CH3OH+H<br>M+CH3OH+H<br>M+K<br>M+K<br>M+K<br>M+ACN+H<br>M+ACN+H<br>M+ACN+H<br>M+ACN+H<br>M+2Na-H<br>M+2Na-H	
5.39	398.3423	C27H45NO2<br>C27H43NO<br>C26H39N<br>C21H44NNaS<br>C25H40O	M+H-H2O<br>M+H<br>M+CH3OH+H<br>M+CH3OH+H<br>M+ACN+H	C27H43NO<br>*CAS:* 80-78-4
5.99	430.2957	C26H41NO5<br>C27H37N5O<br>C25H38N5NaO<br>C22H42N5NaOS<br>C26H39NO4<br>C27H35N5<br>C25H36N5Na<br>C22H40N5NaS<br>C25H37N5<br>C22H41N5S<br>C25H35NO3<br>C22H41N5O<br>C24H36O4<br>C25H32N4<br>C23H33N4Na	M+H-H2O<br>M+H-H2O<br>M+H-H2O<br>M+H-H2O<br>M+H<br>M+H<br>M+H<br>M+H<br>M+Na<br>M+Na<br>M+CH3OH+H<br>M+K<br>M+ACN+H<br>M+ACN+H<br>M+ACN+H	C26H39NO4<br>C27H35N5<br>[M+H]
5.99	465.3098	C26H43NO6<br>C27H39N5O2	M+H-H2O, M+H, M+Na<br>M+H-H2O, M+H, M+Na	C29H40N2O3<br>*CAS:* 112648-68-7, [M+H]

		C25H40N5NaO2<br>C22H44N5NaO2S	M+H-H2O, M+H, M+Na<br>M+H-H2O, M+H, M+Na	C27H42N2O3<br>[M+Na]
5.99	337.2531	C24H34O2<br>C24H32O<br>C23H28	M+H-H2O<br>M+H<br>M+CH3OH+H	C24H32O<br>[M+H]
6.14	352.2404	C24H32O2	M+H-H2O<br>M+H	C21H34ClNO<br>[M+H]
6.93	223.1177	C7H17N3O5<br>C10H18NNaO3	M+H-H2O<br>M+H<br>M+H-H2O<br>M+H	C9H18O6<br>*CAS:* 5391-17-3<br>[M+H]
6.93	372.2679	C24H36O3	M+H-H2O<br>M+H	C22H36FNO<br>*CAS:* 166100-37-4<br>[M+Na]
6.95	426.3213	C24H45NO6<br>C23H42N5NaO2<br>C24H43NO5<br>C23H40N5NaO<br>C23H41N5O<br>C23H39NO4<br>C24H35N5<br>C22H36N5Na<br>C23H46N3Na<br>C23H36N4O<br>C21H37N4NaO	M+H-H2O<br>M+H-H2O<br>M+H<br>M+H<br>M+Na<br>M+CH3OH+H<br>M+CH3OH+H<br>M+CH3OH+H<br>M+K<br>M+ACN+H<br>M+ACN+H	C22H45NO5<br>[M+Na]
7.09	456.3072	C25H44O7<br>C23H45NaO7<br>C24H41N4NaO3	M+H-H2O, M+Na<br>M+H-H2O, M+Na<br>M+H-H2O, M+Na	C26H43NO4<br>[M+Na]
7.28	470.2908	C18H42N5NaO6<br>C18H43N5O6	M+Na<br>M+2Na-H	C26H42ClN3O<br>[M+Na]
10.79	677.2932	C32H47N4NaO8<br>C31H41NO13<br>C30H38N5NaO9<br>C26H49N4NaO12	M+K<br>M+ACN+H<br>M+ACN+H<br>M+2Na-H	C29H46N6O9S<br>[M+Na]

Note: Metabolites generated from the VIP list (a statistically generated list) have a confidence level of >95%. Most probable Metlin match was determined using *m/z* values, retention time, precursor ion, fragmentation patterns, and mass error. These are probable identifications. Additional potential metabolites that differ from those in this list by standard adducts (e.g. Na-H) are not included.
