# Supplementary material for: Metabolomic Diversity and Identification of Antibacterial Activities of Bacteria Isolated From Marine Sediments in Hawai’i and Puerto Rico
Source: Front Mol Biosci. 2020 Feb 25;7:23. doi: 10.3389/fmolb.2020.00023 (PMC7052060; doi:10.3389/fmolb.2020.00023)
Supplement: Supplementary file 3 [file Data_Sheet_2.PDF]

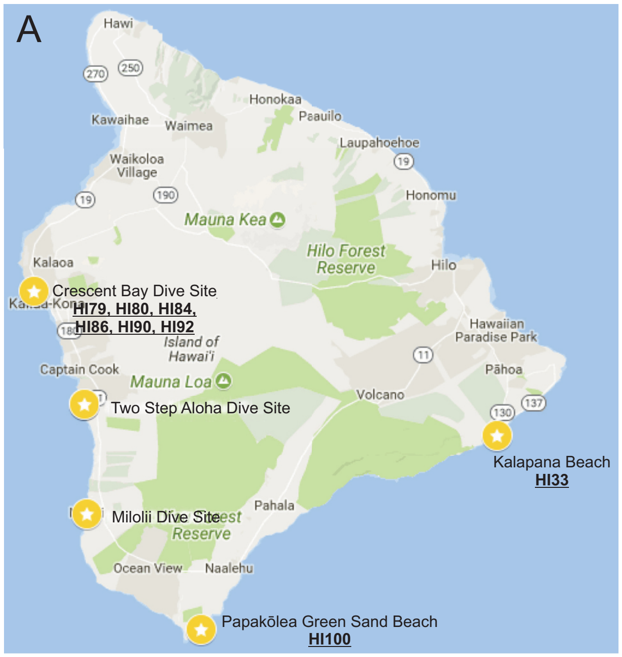

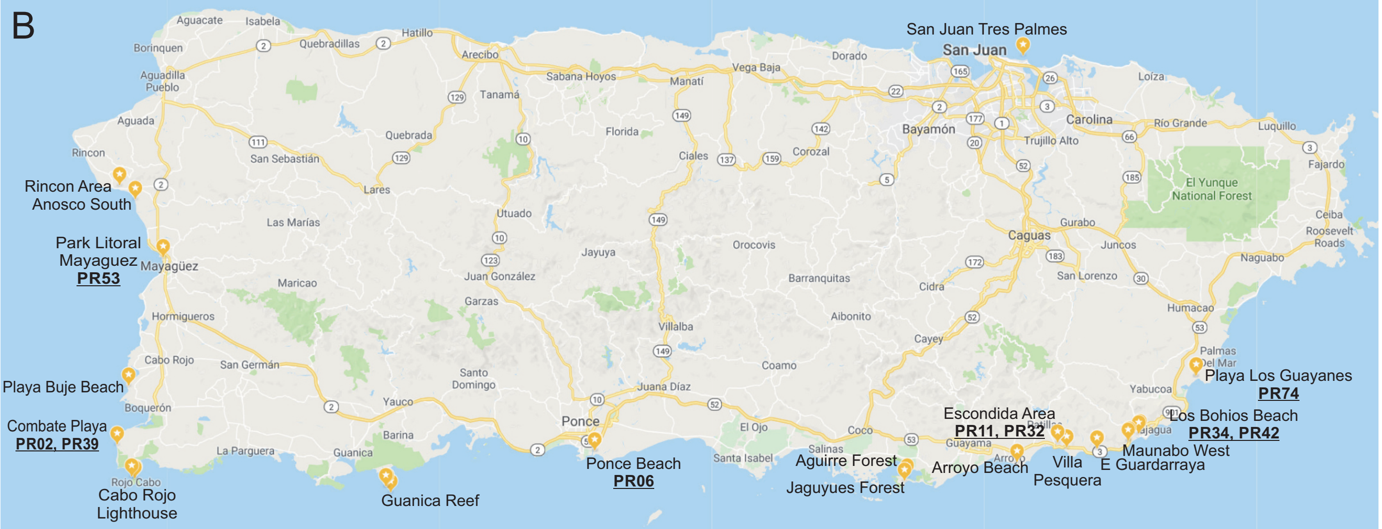

Supplementary Figure 1. Locations of sample collection sites for this investigation. **A**. Map of sample collection sites in Hawai'i. **B.** Map of sample collection sites in Puerto Rico. Stars indicate collection sites, with bolded and underlined code names indicating the bacterial strains included in Figure 3 (phylogenetic tree). Map data: Google.

Table S1. Sample collection locations from Hawaii.

Location and Collection Beach / Dive Site Name	Tube Label	Collection Date/Time	GPS Coordinates of Collection	Depth of Collection	Beach/Site Details/Information
Two step Aloha Dive Site	HI02	2012.06.07 14:51	19°25'26.2"N 155°54'46.7"W 19.423947, -155.912979	7.5 m	Fine white sediment, very popular snorkeling site, sediment in patches between lava-flow-based topology, concrete blocks placed on bottom spelling out ALOHA, collected about 2 m E of blocks
Two step Canyons Dive Site	HI01, HI06	2012.06.07 14:57	19°25'23.8"N 155°54'47.4"W 19.423279, -155.913172	10.5 m	Fine white sediment, very popular snorkeling site, deeper canyons between lava flow ridges, sediment in smaller patch than Aloha site
Crescent Bay Dive Site	HI12, HI13	2012.06.11 14:00	19°40'04.7"N 156°01'41.3"W 19.668094, -156.028191	26 m	Sloping, crescent-shaped bay, at mouth of popular marina, white sediment, collected near echo-location device, white sediment
Milolii Dive Site	HI17	2012.06.09 19:04	19°11'05.9"N 155°54'29.3"W 19.184971, -155.908124	6 m	Very little sediment, mixed with crushed lava rock. Many lava features (columns, arches) at the site, by boat-launch site. Many reef fish around.
Kalapana Beach	HI08	2012.10.06 16:00	19°21'24.2"N 154°58'05.3"W 19.356720, -154.968132	Intertidal Zone	Black sand beach, popular with tourists. Edge of recent lava flows. Very little vegetation around, only a few planted coconut trees in the lava field.
Papakōlea Green Sand Beach	HI14	2012.06.10 14:50	18°56'10.6"N 155°38'45.8"W 18.936288, -155.646066	Intertidal Zone	Green sand beach, unique and popular, but remote and hard to get to.

Table S2. Sample collection locations from Puerto Rico.

Location and Collection Beach / Dive Site Name	Tube Label	Collection Date/Time	GPS Coordinates of Collection	Depth of Collection	Beach/Site Details/Information
San Juan Tres Palmas Beach 1	PR01	2012.11.25 10:00	18°27'11.2"N 66°02'38.8"W 18.453097, -66.044100	Tidal Zone, Wet sand, 10 cm below surface	San Juan Isla Verde, tan find sand, Narrow beach relative to wall, City Beach – urban setting, tourist beach
San Juan Tres Palmas Beach 2	PR02	2012.11.25 10:02	18°27'11.2"N 66°02'38.8"W 18.453097, -66.044100	Tidal Zone, Wet sand, 10 cm below surface	San Juan Isla Verde, tan find sand, Narrow beach relative to wall, City Beach – urban setting, tourist beach
Guanica Reef Dive Site #1	PR03	2012.11.26 07:51	17°55'33.2"N 66°51'54.3"W 17.925899, -66.865070	14 m depth	Reef dive, large reef with open sandy areas in the middle of the reef. Crushed shell, white, light. Edge of reef drops off to great depth.
Guanica Reef Dive Site #2	PR04	2012.11.26 08:02	17°55'32.3"N 66°51'58.3"W 17.925641, -66.866181	14 m depth	Reef dive, large reef with open sandy areas in the middle of the reef. Crushed shell, white, light. 100 m from Guanica Reef #1 site. Edge of reef drops off to great depth.
Cabo Rojo Lighthouse E beach 1	PR05	2012.11.27 11:01	17°56'07.3"N 67°11'25.8"W 17.935359, -67.190491	Tidal Zone, Wet sand, 10 cm below surface	Light – almost white – clean sandy beach. Busy park/tourist beach, near W side of beach, round bay. Fish near shore, tiny, being fed on by Mahi Mahi.
Cabo Rojo Lighthouse E beach 2	PR06	2012.11.27 11:05	17°56'07.3"N 67°11'25.8"W 17.935359, -67.190491	Tidal Zone, Wet sand, 10 cm below surface	Light – almost white – clean sandy beach. Busy park/tourist beach, near W side of beach, round bay. Fish near shore, tiny, being fed on by Mahi Mahi.
Combate Playa Beach 1	PR07	2012.11.27 11:53	17°58'36.6"N 67°12'46.0"W 17.976824, -67.212778	Tidal Zone, Wet sand, 10 cm below surface	Long, wide beach, straight. Pretty secluded, but locals or renters come – few people – no parking. Tan sand.
Combate Playa Beach 2	PR08	2012.11.27 11:54	17°58'36.6"N 67°12'46.0"W 17.976824, -67.212778	Tidal Zone, Wet sand, 10 cm below surface	Long, wide beach, straight. Pretty secluded, but locals or renters come – few people – no parking. Tan sand.

Table S2 (cont.). Sample collection locations from Puerto Rico.

Location and Collection Beach / Dive Site Name	Tube Label	Collection Date/Time	GPS Coordinates of Collection	Depth of Collection	Beach/Site Details/Information
Playa Buye Beach 1	PR09	2012.11.27 12:38	18°02'57.9"N 67°11'55.0"W 18.049417, -67.198611	Tidal Zone, Wet sand, 10 cm below surface	Neighborhood beach, no one around, not much used. Light sand, right up to houses, hardly any beach, hard to find or get to.
Playa Buye Beach 2	PR10	2012.11.27 12:38	18°02'57.9"N 67°11'55.0"W 18.049417, -67.198611	Tidal Zone, Wet sand, 10 cm below surface	Neighborhood beach, no one around, not much used. Light sand, right up to houses, hardly any beach, hard to find or get to.
Park Litoral Mayagüez Beach 1	PR11	2012.11.27 13:33	18°12'22.4"N 67°09'12.3"W 18.206215, -67.153419	Tidal Zone, Wet sand, 10 cm below surface	Large regional park, large palm beach. Not many people around. Trees right at beach. Looks like it is a constructed/fake beach. Sand is dark, almost black. Very urban area.
Park Litoral Mayagüez Beach 2	PR12	2012.11.27 13:34	18°12'22.4"N 67°09'12.3"W 18.206215, -67.153419	Tidal Zone, Wet sand, 10 cm below surface	Large regional park, large palm beach. Not many people around. Trees right at beach. Looks like it is a constructed/fake beach. Sand is dark, almost black. Very urban area.
Anosco South Beach 1	PR13	2012.11.27 13:58	18°16'40.2"N 67°11'23.2"W 18.277832, -67.189764	Tidal Zone, Wet sand, 10 cm below surface	Very wide, long beach, few people, not well taken care of, lots of driftwood. Sand is brown with black intermingled. Trees line the beach, then houses along the Mayagüez city edge. A petrol refinery/plant is 2 km to the south.
Anosco South Beach 2	PR14	2012.11.27 13:59	18°16'40.2"N 67°11'23.2"W 18.277832, -67.189764	Tidal Zone, Wet sand, 10 cm below surface	Very wide, long beach, few people, not well taken care of, lots of driftwood. Sand is brown with black intermingled. Trees line the beach, then houses along the Mayagüez city edge. A petrol refinery/plant is 2 km to the south.

Table S2 (cont.). Sample collection locations from Puerto Rico.

**Location and Collection Beach / Dive Site Name**	**Tube Label**	**Collection Date/Time**	**GPS Coordinates of Collection**	**Depth of Collection**	**Beach/Site Details/Information**
Rincon Area Beach 1	PR15	2012.11.27 14:35	18°17'42.1"N 67°12'36.1"W 18.295026, -67.210039	Tidal Zone, Wet sand, 10 cm below surface	Wide beach, taller than many, medium brown/tan-orange sand, fishermen on beach, built up, expensive neighborhood abuts, Parking area can accommodate many cars, so could be busy beach on nice days. Was raining.
Rincon Area Beach 2	PR16	2012.11.27 14:36	18°17'42.1"N 67°12'36.1"W 18.295026, -67.210039	Tidal Zone, Wet sand, 10 cm below surface	Wide beach, taller than many, medium brown/tan-orange sand, fishermen on beach, built up, expensive neighborhood abuts, Parking area can accommodate many cars, so could be busy beach on nice days. Was raining.
